# Dynamics of PD-1 expression are associated with treatment efficacy and prognosis in patients with intermediate/high-risk myelodysplastic syndromes under hypomethylating treatment

**DOI:** 10.3389/fimmu.2022.950134

**Published:** 2022-08-08

**Authors:** Suxia Geng, Ruohao Xu, Xin Huang, Minming Li, Chengxin Deng, Peilong Lai, Yulian Wang, Ping Wu, Xiaomei Chen, Jianyu Weng, Xin Du

**Affiliations:** Department of Hematology, Guangdong Provincial People’s Hospital, Guangdong Academy of Medical Sciences, Guangzhou, China

**Keywords:** Myelodysplastic syndromes (MDS), programmed death protein 1 (PD-1), programmed death-ligand 1 (PD-L1), programmed death-ligand 2 (PD-L2), hypomethylating agent (HMA)

## Abstract

Hypomethylating agents (HMAs) are widely used in patients with higher-risk MDS not eligible for stem cell transplantation. However, the general response rate by HMAs is lesser than 50% in MDS patients, while the relapse rate is high. Emerging evidence indicates that demethylating effects committed by HMAs may facilitate the up-regulation of a range of immune checkpoints or cancer suppressor genes in patients with MDS, among which the programmed death protein 1 (PD-1) and its ligands are demonstrated to be prominent and may contribute to treatment failure and early relapse. Although results from preliminary studies with a limited number of enrolled patients indicate that combined administration of PD-1 inhibitor may yield extra therapeutic benefit in some MDS patients, identifications of this subgroup of patients and optimal timing for the anti-PD-1 intervention remain significant challenges. Dynamics of immune checkpoints and associated predictive values during HMA-treatment cycles remained poorly investigated. In this present study, expression levels of immune checkpoints PD-1 and its ligands PD-L1 and PD-L2 were retrospectively analyzed by quantitative PCR (Q-PCR) in a total of 135 myelodysplastic syndromes (MDS) cohort with higher-risk stratification. The prognostic value of dynamics of these immune checkpoints during HMA cycles was validated in two independent prospective cohorts in our center (NCT01599325 and NCT01751867). Our data revealed that PD-1 expression was significantly higher than that in younger MDS patients (age ≤ 60) and MDS with lower IPSS risk stratification (intermediate risk-1). A significantly up-regulated expression of PD-1 was seen during the first four HMA treatment cycles in MDS patients, while similar observation was not seen concerning the expression of PD-L1 or PD-L2. By utilizing binary logistic regression and receiver operating characteristic (ROC) models, we further identified that higher or equal to 75.9 PD-1 expressions after 2 cycles of HMA treatment is an independent negative prognostic factor in predicting acute myeloid leukemia (AML) transformation and survival. Collectively, our data provide rationales for monitoring the expression of PD-1 during HMA treatment cycles, a higher than 75.9 PD-1 expression may identify patients who will potentially benefit from the combined therapy of HMA and PD-1 inhibitors.

## Introduction

Myelodysplastic syndromes (MDS) are a heterogeneous group of clonal hematopoietic stem cell diseases characterized by bone marrow failure, dysplasia of myeloid cell linage, and a high risk of acute myeloid leukemia (AML) transformation (1). Hypomethylating agents (HMAs) such as decitabine and azacitidine are the current standard of care for patients with higher-risk MDS (1). Despite prolonged survival achieved when patients respond to HMA, the overall response rate (ORR) remains low, and the duration of response is often transient (2). According to the revised prognostic scoring system of MDS (IPSS-R), median overall survival (OS) ranges from 3.0 years for the intermediate-risk group to 0.8 years for the very high-risk group in MDS, with progression to AML accounting for almost half of deaths (3).

The pathogenesis of MDS remains poorly understood. Studies have revealed the involvement of both hematopoietic cell-intrinsic events (such as age-related mutations) and extrinsic alternations (such as immune deregulation and proinflammatory microenvironment) (4–6). More recently, emerging evidence emphasizes an immune evasion mechanism in the pathogenesis of MDS. Dysfunctional T cells may contribute to the disease progression of MDS and be preferentially associated with a higher risk of AML transformation (7, 8). Negative immune regulatory factors have been proposed to contribute to a protective microenvironment for malignant cells and are associated with a higher risk of AML transformation (9–11).

Immune checkpoint proteins, expressed on different cell subsets with the ability to initiate immune responses either by their activation or inhibition, have been considered a vital part of immune evasion in multiple cancers. The programmed death protein 1 (PD-1) immune checkpoint is considered one of the central mediators of immune tolerance in multiple tumors (12). PD-1 binds two ligands, programmed death-ligand 1 (PD-L1) and PD-L2. PD-L1 is the primary ligand expressed on T and primary B cells, which induces co-inhibitory signals in activated T cells. Furthermore, PD-L1 is expressed in multiple tumor types that deliver negative signals, inhibiting anti-tumor immunity (4). PD-L2 expression is mainly restricted to antigen-presenting cells, such as dendritic cells and macrophages (13). The combined therapy of HMA with PD-1 inhibitors may be of potential therapeutic value in treating patients with higher-risk or relapsed/refractory MDS. Yet another important consideration in the design of an HMA-based combination is the timing of administration of checkpoint inhibitors (14). Evaluation of dynamics of immune checkpoint proteins during HMA treatment cycles may provide rational intervention time points for the combined use of PD-1 inhibitors. However, studies on the dynamics of these checkpoint markers in MDS patients treated with HMA are still limited (15).

To evaluate the dynamics and prognostic value of immune checkpoints PD-1, PD-L1, and PD-L2 in HMA treatment cycles, a total of 135 patients with intermediate/high-risk MDS were enrolled and retrospectively investigated in this present study. Our data identified elevated expression of PD-1 post-HMA treatment may serve as a prognostic marker for inferior survival and AML transformation. Inhibition of the post-HMA elevation of PD-1 may be of potential benefit in higher-risk MDS.

## Materials and methods

### Patients

One hundred thirty-five newly diagnosed and treatment-naïve MDS patients, including 93 males and 42 females, were enrolled in the Guangdong Provincial People’s Hospital from April 2008 to March 2016. For the evaluation of baseline PD-1, PD-L1, and PD-2, expression levels of these immune checkpoints were analyzed in a 102-patient cohort (baseline cohort) under untreated conditions. An additional age- and risk-matched 33-patient cohort from 2 prospective trials serve as the validation cohort to investigate the dynamics and predictive value of the immune checkpoint factors during HMA cycles (16). Treatments for these patients are azacitidine 75mg/m^2^/day subcutaneously (SC) for 7 days every 28 days (NCT01599325, n=16) and decitabine 15mg/m^2^ as a continuous intravenous infusion within 3 hours, repeated every 8 hours for 3 consecutive days (NCT01751867, n=17). Written informed consent was obtained from all patients. The present retrospective study was approved by the Institutional Ethics Committee of Guangdong Provincial People’s Hospital. Diagnoses were conducted according to the French-American-British classification and re-classified according to the 2016 edition of WHO classification of myeloid neoplasms and acute leukemia. The median age of the enrolled patients was 60 (15-84) years. All patients were classified as the intermediate/high-risk group according to the international prognostic scoring system (IPSS) (17). As the revised edition of the international scoring system (IPSS-R) has re-classified the prognosis of MDS into 5 prognosis-based stratifications (18), we re-calculated the scores of MDS patients according to each edition of IPSS systems and compared risk-based stratifications. Results revealed that the utilization of IPSS-R did not significantly change the intermediate/high-risk entity of these enrolled MDS patients (**Supplemental Table 1**). Thus, the IPSS- stratifications were kept and utilized in the subsequent risk-based analysis. Karyotypes were classified according to the new comprehensive cytogenetic scoring system for primary MDS and oligoblastic acute myeloid leukemia (19). All baseline characteristics, including sex ratio, median age, bone marrow (BM) blast percentage, WHO classification, and IPSS risk stratification, remained similar between the baseline and the validation cohort (**Table 1**).

**Table 1:** Baseline characteristics of enrolled patients.

	Baseline cohort (n=102)	Validation cohort (n=33)	*P* value
**Sex, n (%)**			0.58
Male Female	69 (66.7%) 33 (32.3%)	24 (72.7%) 9 (27.3%)	
**Median age (year)**	60 (15-84)	61 (38-73)	0.67
**2016 WHO classification, n (%)**			0.61
RAEB1 RAEB2 MLD	42 (41.2%) 25 (24.5%) 35 (34.3%)	16 (48.5%) 12 (36.4%) 5 (15.2%)	
**Hemoglobin (g/L)**	72 (49-116)	68 (42-49)	0.34
**Leukocyte count (10^9^/L)**	2.4 (0.69-11.90)	2.3 (0.88-11.75)	0.82
**Platelet count (10^9^/L)**	71 (11-391)	68 (14-340)	0.51
**Neutrophil count (10^9^/L)**	1.07 (0.22-11.31)	0.97 (0.35-10.03)	0.61
**Blast% (bone marrow)**	5% (1%-18%)	5% (1%-16.5%)	0.36
**Cytogenetics, n (%)**			0.82
Good Intermediate Poor Very poor unassessable	59 (57.8%) 28 (27.5%) 6 (6.0%) 1 (1.0%) 8 (8.0%)	19 (57.6%) 8 (24.2%) 2 (6.1%) 1 (3.0%) 3 (9.1%)	
**IPSS risk group, n (%)**			0.42
Int-1 Int-2 High	46 (45.1%) 32 (31.4%) 24 (23.5%)	11 (33.3%) 13 (39.4%) 9 (27.3%)	

### RNA extraction and cDNA synthesis

Whole bone marrow mononuclear cells (MNCs) were collected from patients at the time points of pre-treatment, after the 2^nd^ (C2), the 4^th^ HMA cycle (C4), and the 6^th^ HMA treatment cycle (C6). Total RNA was extracted with TRIzol (Life Technologies) according to the manufacturer’s recommendations. The quality of extracted RNA was analyzed using a 0.8% agarose gel stained with Goldview. RNA (~1μg) was synthesized into the first single-strand cDNA with random hexamer primers using the PrimeScript™ RT Reagent Kit (TaKaRa) for subsequent quantitative PCR assays.

### Quantitative PCR (Q-PCR)

Quantification of PD-1, PD-L1, and PD-L2 transcripts was performed by real-time PCR (TaqMan) with the ABI 7500 Sequence Detection System (Applied Biosystems, Foster City, CA) as previously described (20). The internal control gene ABL1 was used for normalization of the Q-PCR results to compensate for variations in the quality and quantity of RNA and cDNA (21). PD-1, PD-L1, and PD-L2 Q-PCR primers and probes were designed by Primer Express software and synthesized by Life Technologies (**Supplemental Table 2**). The amplification efficiency of the primers and probes was determined. ABL1 plasmids standard (1×10^6^, 1×10^5^, 1×10^4^, 1×10^3^, and 1×10^2^ copies/μl) were made. The amplification efficiency of the target genes was close to that of the ABL1 reference gene; thus, they shared a set of standards.

Q-PCR reactions for the cDNA samples, DNA standards, and water as negative control were conducted in a total volume of 20 μL, including 10 μL 2× FastStart Universal Probe Master (ROX) (Roche, Mannheim, Germany), 300 nM of each primer, and 200 nM probe. The thermal cycler parameters were as follows: 2 minutes at 50°C, 10 minutes at 95°C, and 45 cycles of 95°C for 15 seconds and 62°C for 1 minute. The expression levels of the target genes are indicated as “(copy number of the target gene/copy number of the internal reference*100) %” with comparisons between different samples. All PCR assays were performed in duplicate and reported as means.

### Flow cytometry

Cell surface staining for flow cytometry was performed using the following antibodies: CD3-AF700 (clone UCHT1, BD), CD4-APC-H7 (clone RPA-T4, BD), CD8-APC-H7 (clone SK1, BD) and PD-1-BV421 (clone EH12.2H7, Biolegend). Isotype-matched antibodies, labeled with the proper fluorochromes, were used as negative controls. Cells were analyzed using a BD Fortessa flow cytometer (BD Biosciences), and data analysis was performed with Flowjo 10.6 software as previously described (22).

### Targeted gene sequencing

Targeted gene sequencing of a 13-gene panel of hotspot mutations was performed using whole bone marrow mononuclear cells (MNCs) at diagnosis. These hot mutations including TET2, TP53, DNMT3A, and ASXL1 were listed. (**Supplemental Table 3**).

### Statistical analysis

All data were analyzed using SPSS software (version 19.0; IBM Corp.) and presented with mean ± SEM. Differences in PD-1, PD-L1, and PD-L2 expression between two groups were analyzed using Student’s t-test or Mann-Whitney u-test. Differences among multiple groups were determined by one-way or two-way ANOVA followed by Tukey’s *post hoc* test. For comparisons between paired samples, paired t-test was applied. Spearman correlation analysis was used to analyze correlations. The Wilcoxon signed-rank test was used to compare data between two paired groups. Receiver operating characteristic (ROC) curves were used to evaluate factors’ sensitivity and specificity in predicting AML transformation events. A binary logistic regression model was used to investigate the predictive value of factors in predicting AML transformation events. A p-value lower than 0.05 was considered statistically significant.

## Results

### Baseline and subgroup expression of PD-1, PD-L1, and PD-L2 in MDS

**Figure 1:**
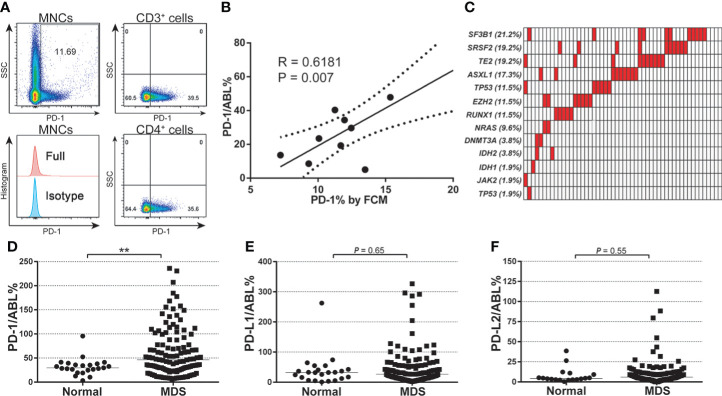
Baseline expression of PD-1, PD-L1, PD-L2, and mutational characteristics of MDS patients. **(A)** FCM detection for the expression of PD-1 protein in each gated cell subset in BM-MNC samples from patients with MDS (n=9). **(B)** Pearson analysis between the PD-1 gene expression (qPCR value) and protein levels in patients with MDS (n=9, R = 0.6181, P = 0.007). **(C)** Mutational profile of a 13-gene panel of hotspot mutations (**Supplemental Table 3**) in MDS patients (n=52). **(D)** Normalized baseline expression of PD-1 in the bone marrow samples from normal individuals (n=23) and MDS patients (n=102). **(E)** Normalized baseline expression of PD-L1 in the bone marrow samples from normal individuals (n=23) and MDS patients (n=102). **(F)** Normalized baseline expression of PD-L2 in the bone marrow samples from normal individuals (n=18) and MDS patients (n=102). Results were presented as mean ± SEM of independent cases. *P < 0.05. **P < 0.01. ***P < 0.001.

Of 135 enrolled patients in this study, 58 patients (58/135, 43.0%) were diagnosed with refractory anemia with excess blast 1 (MDS-EB1), 37 patients (37/135, 27.4%) with MDS-EB2, and 40 patients (40/135, 29.6%) with MDS with multilineage dysplasia (MDS-MLD). The median age of enrolled patients was 60 (15-84) years. All patients were assessed and were classified into the intermediate/high-risk group according to the international prognostic scoring system (IPSS) (17). To test the reliability of the Q-PCR method in investigating checkpoint expression in BM, paired Q-PCR and flow cytometry assays for PD-1 were performed using MNC samples at diagnosis from nine patients with MDS. A median of 11.69% (7.22%-20.25%) MNCs were positive for PD-1 expression by flow cytometry (FCM) assays (**Figure 1A**), while the median Q-PCR expression for PD-1 in these samples was 29.72 (5.06-47.88). Pearson’s correlation analysis indicated that PD-1 expression levels by FCM assays correlated with those from Q-PCR assays (R = 0.6181, P = 0.007) (**Figure 1B**). Thus, these results confirmed the feasibility of the Q-PCR method in evaluating immune checkpoint expression in MNC samples. For mutation profiling, targeted gene sequencing of a 13-gene panel of hotspot mutations was performed using whole bone marrow mononuclear cells (MNCs) at diagnosis. These hot mutations including TET2, TP53, DNMT3A, and ASXL1 were listed. (**Supplemental Table 3**). Generally, a high frequency of hotspot mutations was detected in 92.3% (48/58) patients, with the most frequent mutations seen in SF3B1 (21.2%), SRSF2 (19.2), TET2 (19.2%), and ASXL1 (17.3%) (**Figure 1C**). By profiling the expression levels of PD-1, PD-L1, and PD-L2 in MNCs from MDS and normal individuals, a significantly elevated expression of PD-1 was seen in MDS samples compared with normal samples (58.77 ± 3.820 vs. 31.95 ± 3.692, P = 0.007) (**Figure 1D**). In contrast, the expression of PD-L1 and PD-L2 in MDS patients was not significantly different from healthy individuals (**Figures 1E, F**).

**Figure 2:**
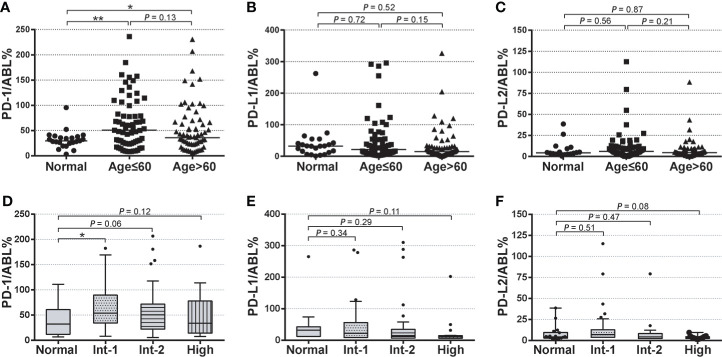
Baseline expression of PD-1, PD-L1, and PD-L2 in age-based and risk-based MDS subgroups **(A–C)** Normalized baseline expression levels of PD-1, PD-L1, and PD-L2 in healthy individuals, young MDS (age ≤ 60 years), and older MDS (age>60 years). **(D–F)** Normalized baseline expression levels of PD-1, PD-L1, and PD-L2 in healthy individuals and patients with intermediate-1, intermediate-2, and high-risk stratification. Results were presented as mean ± SEM of independent cases. *P < 0.05. ***P < 0.001.

Next, PD-1, PD-L1, and PD-L2 expression levels in MDS subgroups were investigated. BM samples from both the young MDS cohort (age ≤ 60 years) and older MDS cohort (age > 60 years) displayed significantly higher PD-1 expression than that in the normal cohort, while those young MDS patients were associated with a slightly higher PD-1 expression than in older MDS patients (64.49 ± 5.592 vs. 52.91 ± 5.147, P = 0.13) (**Figure 2A**). Expression levels of PD-L1 and PD-L2 remained similar across age-based subgroups in the MDS cohorts (**Figures 2B, C**). For expression levels of these immune checkpoints in IPSS-based MDS subgroups, PD-1 expression was significantly higher in the intermediate-1 risk MDS than that in normal samples (59.17 ± 7.484 vs. 31.95 ± 3.692, P = 0.02), and slightly higher in the intermediate-2 (48.38 ± 5.862 vs. 31.95 ± 3.692, P = 0.06) and high-risk group (50.67 ± 12.91 vs. 31.95 ± 3.692, P = 0.12) (**Figure 2D**). Expression levels of PD-L1 and PD-L2 were not significantly up-regulated in MDS patients with intermediate-1 or intermediate-2 risk groups. Interestingly, a trend of lower expression of PD-L1 (23.35 ± 10.84 vs. 31.95 ± 3.692, P = 0.11) and PD-L2 (3.523 ± 0.479 vs. 7.847 ± 2.286, P = 0.08) were seen in those BM samples from MDS patients from the high-risk group.

### Dynamics of PD-1, PD-L1, and PD-L2 expression in HMA treatment cycles

To elucidate the dynamics of PD-1, PD-L1, and PD-L2 expression and potential predictive value during HMA treatment cycles, expression levels of these immune checkpoints at timepoints of pre-treatment (baseline), after the 2^nd^ cycle (C2), 4^th^ cycle (C4) and 6^th^ cycle (C6) of HMA treatment were analyzed. Furthermore, treatment response and survival data were extracted and analyzed in an additional 33-patient cohort of intermediate/high-risk MDS from two clinical trials (NCT01599325 and NCT01751867). In the validation cohort, 51.5% of patients (17/33) were treated with decitabine, while 48.5% (16/33) received azacitidine. HMA dosages and treatment schedules could be seen in our previous report (16). The median number of treatment cycles was 12 (3-21), and 57.6% of patients (19/33) acquired at least 1 clinical response to HMA (CR/mCR/HI) according to the IWG 2006 criteria (23).

**Figure 3:**
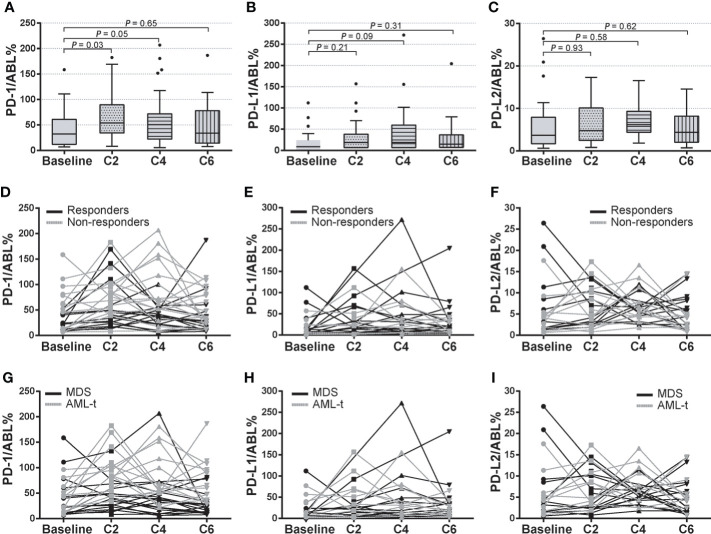
Dynamics of PD-1, PD-L1, and PD-L2 expression in HMA treatment cycles. **(A–C)** Pre-treatment and post-treatment expression of PD-1, PD-L1, and PD-L2 in HMA treatment cycles in MDS patients who received at least 2-cycle of HMA treatment. **(D–F)** Dynamic expression of PD-1, PD-L1, and PD-L2 in HMA treatment cycles in HMA responders and HMA non-responders. **(G–I)** Dynamic expression of PD-1, PD-L1, and PD-L2 in HMA treatment cycles in MDS patients with or without AML transformation event. Results were presented as mean ± SEM of independent cases.

Compared with the expression at pre-treatment condition, PD-1 levels significantly increased after the first 2 cycle (C2) of HMA treatment (66.38 ± 7.709 vs. 42.74 ± 7.405, P = 0.03), then gradually decreased after the 4^th^ (47.58 ± 7.408 vs. 61.23 ± 9.304, P = 0.05) and 6th (47.58 ± 7.408 vs. 42.74 ± 7.405, P = 0.65) HMA cycles (**Figure 3A**). Similar trends of up/down-regulated PD-L1 and PD-L2 were also seen in these MDS patients, while these differences did not reach statistical significance during HMA treatment cycles (**Figures 3B, C**). To investigate whether PD-1, PD-L1, and PD-L2 dynamics were associated with treatment efficacies, MDS patients were classified as HMA responders or HMA non-responders according to the IWG 2006 criteria (23). Expression of these markers at time points of pre-treatment (baseline), C2, C4, and C6 were analyzed and compared between the two groups (**Figures 3D–F**). Generally, the expression of PD-1, PD-L1, and PD-L2 fluctuated through treatment cycles. Expression of PD-1 increased in most HMA non-responders after the 2^nd^ treatment (12/14, 85.7%) and remained higher than that in those HMA responders (78.58 ± 8.302 vs. 55.28 ± 6.340, P = 0.06). After the 4^th^ cycle of HMA treatment, PD-1 expression decreased in most HMA responders (13/19, 68.4%), while the PD-1 expression remained at elevated levels in half of the HMA non-responders (7/14, 50.0%) (**Figure 3D**). However, no difference was observed concerning the expression of PD-L1 or PD-L2 between HMA responders and non-responders through HMA cycles (**Figures 3E, F**).

### PD-1 dynamics in HMA treatment cycles were associated with the risk of AML transformation

Patients with higher-risk MDS faced a higher risk of AML transformation (1, 18). In this study, 17 patients progressed to AML in the validation cohort (17/33, 51.5%), with a median leukemia-free survival (LFS) of 24.0 months. Subgroup analysis was performed in patients with AML transformation (AML-t, n=17) and patients without AML transformation (MDS, n=16). Expression of these markers at time points of baseline, C2, C4, and C6 was analyzed. The median expression of PD-1 significantly increased at C2 (76.39 ± 16.419 vs. 46.12 ± 12.315, P = 0.04) and C4 (71.22 ± 24.915 vs. 46.12 ± 12.315, P = 0.05) than the baseline PD-1 expression in the AML-t subgroup, then decreased at C6. By utilizing paired t-test analysis between subgroups, the AML-t group displayed significantly higher expression levels of PD-1 at C2 (81.92 ± 17.482 vs. 54.21 ± 14.315, P = 0.03) and C4 (74.31 ± 21.294 vs. 43.987 ± 11.411, P = 0.05) than the that in the non-transformed group (**Figure 3G**). No correlation was seen between the incidence of AML transformation and the expression of PD-L1 or PD-L2 in the HMA treatment cycles (**Figures 3H, I**). These data indicated a potential prognostic value of post-HMA dynamics of PD-1 expression in predicting AML transformation events in higher-risk MDS patients.

### Up-regulated PD-1 after the 2^nd^ treatment cycle predicts long-term survival after HMA treatment

**Figure 4:**
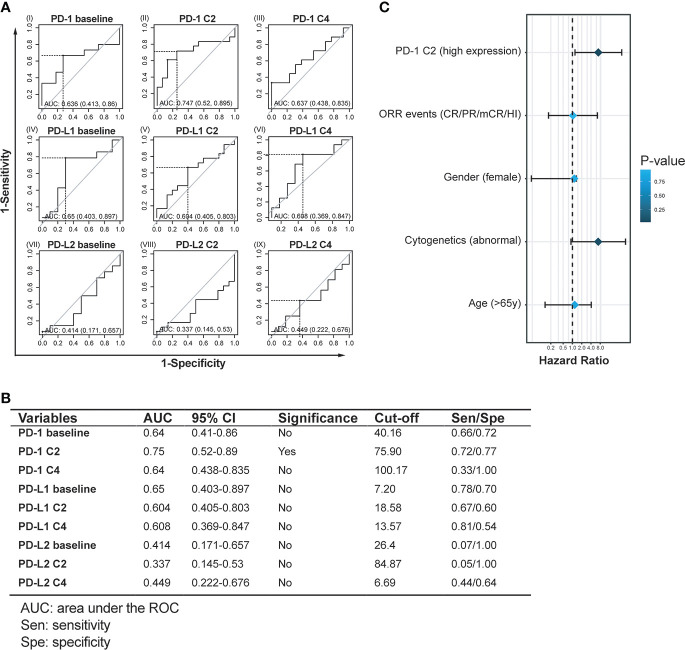
Assessment of objective cut-off and prognostic value of PD-1, PD-L1, and PD-L2 expression in HMA treatment cycles. **(A, B)** Receiver operating characteristic (ROC) curves and statistics of PD-1, PD-L1, and PD-L2 expression at time points predict AML transformation. **(C)** Binary logistic regression analysis for factors to predict AML transformation in MDS patients treated with HMA.

Next, receiver operating characteristic (ROC) models were further utilized to validate the sensitivity and specificity of expression levels of immune checkpoints in predicting AML transformation events. By enrolling expression levels of these checkpoints at baseline, C2, and C4, the specificity and sensitivity of each factor in predicting AML transformation events were calculated and displayed. Generally, most checkpoints failed to display values predicting AML transformation events (**Figures 4A, B**). However, PD-1 expressions at C2 were associated with a significant value to predicted AML transformation, which yielded an area under the ROC curve (AUC) of 0.747 (0.520-0.895), with a cut-off value of 75.9 and a sensitivity/specificity ratio of 0.72/0.77 (P < 0.05) (**Figure 4B**). By using the calculated PD-1 cut-off value of 75.9 at C2 as a factor and re-classifying MDS patients into high PD-1 expression group (≥75.9, n=17) and low PD-1 expression group (<75.9, n=16), a binary logistic regression analysis enrolling PD-1 C2 expression, ORR, gender cytogenetics, and age was performed. Generally, high PD-1 expression at C2 was significantly associated with a higher risk of AML transformation (HR:6.919; 95%CI:1.213-39.47, P=0.03). Meanwhile, abnormal cytogenetics also predicted the AML transformation events in the present MDS cohort (HR: 6.863; 95%CI: 0.895-52.607, P=0.06), while the factors of ORR events (HR: 1.045; 95%CI: 0.169-6.447, P=0.962), female gender (HR: 1.151; 95%CI: 0.047-1.341, P=0.896) and elder age (HR: 1.191; 95%CI: 0.130-4.101, P=0.845) did not reach a statistic significance in the logistic regression model (**Figure 4C**).

**Figure 5:**
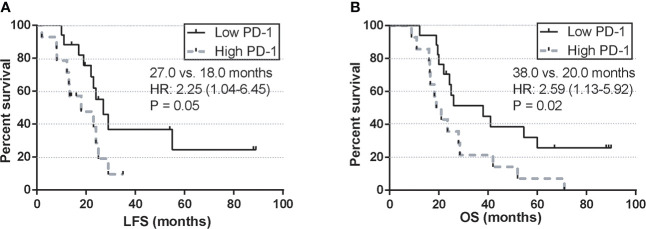
Univariate survival analysis of LFS and OS by the post-treatment PD-1 expression. **(A)** LFS by PD-1 expression at C2 in MDS patients treated with HMA. **(B)** OS by PD-1 expression at C2 in MDS patients treated with HMA.

To further validate the long-term prognostic value of PD-1 after the 2^nd^ HMA treatment cycle, a univariate survival analysis was performed between the high PD-1 expression group and the low PD-1 expression group. Four patients were still alive at the last follow-up, with a median follow-up of 23.4 months in the whole cohort. Median leukemia-free survival (LFS) was 27.0 months in the low PD-1 group, whereas in the high PD-1 group was 18.0 months (HR: 2.25; 95%CI: 1.04-6.45; log-rank test, P=0.05) (**Figure 5A**). For overall survival, 2-year OS in the low PD-1 group was 93.8% (15/16), whereas in the high PD-1 group was 88.2% (15/17). Those MDS patients in the low PD-1 group were associated with significantly longer estimated OS than that in the high PD-1 group (38.0 vs. 20.0 months; HR:2.590; 95%CI: 1.13-5.92, P = 0.02) (**Figure 5B**).

## Discussion

The treatment response/resistance mechanisms after HMA cycles were not fully understood until now. Existing data indicated that dysregulated gnomic-wide methylation is closely involved in the development and progression of MDS (24). Thus, demethylation and reactivation of silenced tumor-suppressing genes are initially considered pivotal mechanisms during the treatment cycles of HMA and other HMA-based treatment schemes (25, 26). With emerging evidence indicating a wider range of cellular/molecular regulations by HMAs, induced expression of tumor antigens (27) and enhancement of effective T cells (28) may represent parallel mechanisms. However, despite prolonged survival in patients who have responded to HMA, the overall response rate (ORR) to HMAs remains low (~50%), and the duration of treatment response is often transient (2). Loss of response frequently happens within 2 years after the first administration of HMAs, with no standard-of-care options for patients after treatment failure. Expected survival for these patients remains dismal (29).

On the other hand, emerging evidence indicates a “side-effect” of HMAs underlying treatment failure and disease progression. Based on updated concepts, HMAs demethylate a range of immune checkpoints with negative prognostic values in multiple cancers (30–32). Enhanced expression of PD-1, PD-L1, PD-L2, CTLA-4, and other immune checkpoints after HMA treatment potentially contributes to an immunosuppressive bone marrow/peripheral environment in MDS patients (15, 33). Moreover, a recent study by Liu, Y.C., et al. revealed that HMAs strikingly enhance the expression of SALL4 (a well-described oncogene) by demethylation in its CpG island within the 5’ untranslated region in a group of MDS. This demethylating effect on SALL4 was then confirmed to associate with an inferior clinical outcome (34–36).

In this context, many combined therapies using HMA with novel drugs were designed for long-term synergistic effects and prolonged survival in treating MDS (26, 37). These combinations included HMA plus immune checkpoint inhibitors (anti-PD-1/PD-L1) (38, 39), HMA plus histone deacetylase inhibitors (HDACi) (40, 41), and HMA plus immunosuppressive agent (lenalidomide) (42, 43) and others. Combining HMA with immune checkpoint inhibitors is designed primarily to sensitize the antitumoral immune response of these therapies. However, although some HMA-based combined therapies have demonstrated a favorable response rate in patients with higher-risk MDS, survival benefit was not achieved in these trials. At the same time, non-neglectable toxicities were frequently noted (38, 39). A recent head-to-head study by Zeidan, A.M., et al. revealed the combination of azacitidine plus durvalumab (a PD-L1 inhibitor) leads to up to 89.5% grade 3-4 hematologic adverse events (AEs) in higher-risk MDS, while the incidence of grade 3-4 AEs remains 68.3% in patients treated with single azacitidine (39). Thus, a more rationally designed medication timing and dosage of these combinations will be especially important. Evaluation of baseline and dynamic expression of immune checkpoints during HMA treatment cycles may provide evidence for patient selection and rational timing for an anti-PD-1 intervention. However, the dynamics of immune checkpoints in HMA treatment cycles remain largely uninvestigated in patients with MDS (15), especially in those patients with higher IPSS stratification.

In previous studies, Yang et al. showed that the mRNA expression of PD-1, PD-L1, and PD-L2 was increased in CD34+ cells and peripheral blood mononuclear cells from MDS patients (15). Kondo et al. found that PD-1 expression on CD3+, CD4+, and CD8+ T cells was significantly increased in MDS patients (44). Similar to these reports, our data revealed a significantly elevated baseline expression of PD-1 in the bone marrow of patients with MDS (**Figure 1A**). However, expression levels of PD-L1 and PD-L2 in the MDS cohort remained similar to the normal individuals (**Figures 1E, F**). In contrast with the observation from Yang et al. (15), our result showed that PD-1 expression was slightly higher in high-risk MDS patients of younger age (**Figure 2A**), and the expression level of PD-L1 and PD-L2 remained similar between age-based MDS subgroups (**Figures 2B, C**).

Interestingly, although the expression levels of PD-1 in MDS were generally upregulated, it seemed that there were discrepant expression levels of immune checkpoints within risk-based subgroups in higher-risk MDS. Patients with intermediate-1 risk stratification always displayed with highest median expression levels of PD-1, PD-L1, and PD-L2. In contrast, the expression levels decreased when the IPSS risk score increased and remained lowest in the high-risk MDS (**Figures 2D–F**). A recent study has revealed time- and dose-dependent upregulation of immune checkpoints in CD34+ cells *in vitro* (15). Similar to this observation, our data indicated a post-HMA up-regulation of PD-1, PD-L1, and PD-L2 in MDS patients. Median expression of PD-1 was significantly up-regulated after 2 cycles of HMA treatment, then gradually decreased during continuous HMA treatment (**Figure 3A**). The potential mechanism of these immune checkpoints’ up-regulation may be attributed to the demethylation effect by HMA on the transcripts (15, 33). In contrast, the mechanism of decrease of these markers after continuous administration of HMA remains unknown.

Next, clinical correlations between dynamics of immune checkpoints and clinical outcomes were seen by monitoring the expression of PD-1, PD-L1, and PD-L2 in each MDS patient. Unlike the previous studies, which reported a clinical correlation of baseline expression of PD-1 in MDS patients (15), our data indicated that only the upregulation of PD-1 after the 2^nd^ cycle of HMA treatment was associated with inferior ORR in higher-risk MDS patients. At the same time, similar observations were not seen concerning the baseline expression of PD-1 (**Figure 3D**). For long-term survival, MDS patients with intermediate/high-risk stratification faced a higher risk of AML transformation and AML-related mortality (3). Our data indicated that those MDS patients who eventually progressed to AML displayed a significantly higher PD-1 expression of PD-1 after the 2^nd^ cycle of HMA treatment (**Figure 3G**). To further elucidate the predictive value of PD-1, PD-L1, and PD-L2 at each timepoint in HMA treatment cycles, receiver operating characteristic (ROC) curves were used to evaluate the potential sensitivity and specificity of factors in predicting AML transformation event. Similar to the results above, the baseline expression of PD-1, PD-L1 and PD-L2 was not associated with a significant value in predicting AML transformation events. Only the PD-1 expression after the 2^nd^ HMA treatment was associated with significant specificity and sensitivity in predicting AML transformation (**Figure 4B**). The optimal cut-off of the PD-1 expression after the 2^nd^ HMA treatment cycle was compromised at 75.9, with a sensitivity/specificity of 0.72/0.77. An additional binary logistic regression model further validated the prognostic value of the 75.9 cut-off of PD-1 (**Figure 4C**). At last, by binarily grouping MDS patients into the low PD-1 group and high PD-1 group using this calculated cut-off, significant inferior LFS and OS were confirmed in the high PD-1 group (**Figure 5**).

In summary, this present study identified discrepant expression profiles of immune checkpoints in age- and risk-based MDS subgroups. Our data provide detailed dynamics of up-regulation of PD-1 after HMA treatment and further identified the ≥75.9 PD-1 expression as an independent negative prognostic factor in higher-risk MDS patients. At last, evaluation of the bone marrow PD-1 expression after the 2^nd^ cycle of HMA treatment may identify patients who will benefit from the combined therapy of HMA and PD-1 inhibitors.

## Data availability statement

The original contributions presented in the study are included in the article/**Supplementary Material**. Further inquiries can be directed to the corresponding authors.

## Ethics statement

The studies involving human participants were reviewed and approved by institutional ethics committee of Guangdong Provincial People’s Hospital. The patients/participants provided their written informed consent to participate in this study.

## Author contributions

XD, JW, SG, and RX designed the research, participated in data analysis and interpretation, and drafted the manuscript. SG, XH, RX, YW, and PL performed the Real-time PCR, flow cytometry, and targeted gene sequencing analysis. CD, ML, XC, XH, and PW contributed to patients and provided blood samples. All authors contributed to the article and approved the submitted version.

## Funding

This study was supported by the National Natural Science Foundation of China (82070128, 82070176), Guangdong Natural Science Foundation (2021A1515011436, 2019A1515010094), and Guangdong Provincial Science and Technology Projects (2017B020230004).

## Conflict of interest

The authors declare that the research was conducted in the absence of any commercial or financial relationships that could be construed as a potential conflict of interest.

## Publisher’s note

All claims expressed in this article are solely those of the authors and do not necessarily represent those of their affiliated organizations, or those of the publisher, the editors and the reviewers. Any product that may be evaluated in this article, or claim that may be made by its manufacturer, is not guaranteed or endorsed by the publisher.
