# Supplementary material for: Dynamics of PD-1 expression are associated with treatment efficacy and prognosis in patients with intermediate/high-risk myelodysplastic syndromes under hypomethylating treatment
Source: Front Immunol. 2022 Aug 8;13:950134. doi: 10.3389/fimmu.2022.950134 (PMC9393298; doi:10.3389/fimmu.2022.950134)
Supplement: Supplementary file 1 [file DataSheet_1.docx]

**Supplemental Table 1. IPSS and IPSS-R risk stratifications in the validation cohort**

| IPSS stratification (n=33) | Risk groups | IPSS-R stratification (n=33) | Risk groups |
| --- | --- | --- | --- |
| Low  Int-1  Int-2  High | 0  11 (33.3%)  13 (39.4%)  9 (27.3%) | Very low  Low  Intermediate  High  Very high | 0  0  14 (42.4%)  13 (39.4%)  6 (18.2%) |

**Supplemental Table 2. The primer and probe sequences of PD-1, PD-L1, and PD-L2 in Q-PCR analysis**

| Primer/Probe | Sequence (5’ to 3’) |
| --- | --- |
| PD-1-F | TCGGAGAGCTTCGTGCTAAAC |
| PD-1-R | GGAAGGCGGCCAGCTT |
| PD-1-P | FAM-ATGAGCCCCAGCAACCAGACGG-TAMRA |
| PD-L1-F | GGTGCCGACTACAAGCGAAT |
| PD-L1-R | TGACTGGATCCACAACCAAAATT |
| PD-L1-P | FAM-TCAATGCCCCATACAACAAAATCAACCAA-TAMRA |
| PD-L2-F | CATCCAACTTGGCTGCTTCA |
| PD-L2-R | TTAGGGCTATCACTGTGGCTATGA |
| PD-L2-P | FAM-CATCCCCTTCTGCATCATTGCTTTCATT-TAMRA |

**Supplemental table 3. Sequencing panel of hotspot mutations**

| NRAS | CBL | JAK2 |
| --- | --- | --- |
| TP53 | RUNX1 | DNMT3A |
| TET2 | IDH1 | IDH2 |
| EZH2 | ASXL1 | SRSF2 |
| SF3B1 |  |  |
